# Recruited mast cells in the tumor microenvironment enhance bladder cancer metastasis via modulation of ERβ/CCL2/CCR2 EMT/MMP9 signals

**DOI:** 10.18632/oncotarget.5467

**Published:** 2015-11-05

**Authors:** Qun Rao, Yuan Chen, Chiuan-Ren Yeh, Jie Ding, Lei Li, Chawnshang Chang, Shuyuan Yeh

**Affiliations:** ^1^ Department of Gynaecology and Obstetrics, Tongji Medical College/Hospital, Huazhong University of Science and Technology, Wuhan, China; ^2^ Sex Hormone Research Center, Department of Urology, Tongji Medical College/Hospital, Huazhong University of Science and Technology, Wuhan, China; ^3^ George Whipple Lab for Cancer Research, Departments of Urology and Pathology, University of Rochester Medical Center, Rochester, NY, USA

**Keywords:** tumor associated immune cells, ERβ antagonist, oncology, carcinogenesis

## Abstract

Early clinical studies suggested that infiltrating mast cells could be associated with a poor outcome in bladder cancer (BCa) patients. The mechanisms of how mast cells influence the BCa progression, however, are unclear. Using the human clinical BCa sample survey and *in vitro* co-culture systems, we found BCa cells could recruit more mast cells than the surrounding non-malignant urothelial cells. The consequences of this better recruitment of mast cells toward BCa cells could then enhance BCa cell invasion. Mechanism dissection revealed that the enhanced BCa cell invasion could function via up-regulation of the estrogen receptor beta (ER&beta;) in both mast cells and BCa cells, which resulted in the increased CCL2/CCR2/EMT/MMP9 signals. Using the pre-clinical mouse BCa model, we further validated the mast cell-promoted BCa invasion. Interruption of the newly identified ER&beta;/CCL2/CCR2/EMT/MMP9 pathway via either ER&beta;-siRNA, ER&beta; antagonist PHTPP, or CCR2 antagonist can effectively reverse the mast cell-enhanced BCa cells invasion. Together, our finding could lead to the development of an alternative new therapeutic approach to better treat BCa metastasis.

## INTRODUCTION

Bladder cancer (BCa) is one of the most prevalent malignancies of the urinary tract with the global prevalence at approximately 1 million annually and the case number is steadily increasing [1]. While most BCa are non-muscle invasive tumors, many of them may relapse and progress to invasive cancer, and 5% to 20% of superficial tumors may eventually develop into muscle-invasive disease despite different treatments [2].

There are two major types of estrogen receptors (ERs), ER alpha (ERα) and ER beta (ERβ), mediating estrogen effects in various tissues [3–7]. Estrogens and their ERs its estrogen receptors (ERs) have been implicated as playing important roles in urological diseases [8–13] and in BCa [14–17]. Using the ER gene knockout strategy in carcinogen-induced BCa models, results from the preclinical animal models proved the differential roles of ERα and ERβ in different stages of BCa progression. Comparing the immunohistochemical staining of the ERα and ERβ protein expression, the ERβ level is reported to be more predominant in clinical BCa samples [18–21]. Importantly, a growing body of evidence showed that ERβ is a biomarker linked to the poor worse oncologic outcomes in BCa patients [15, 20–22].

Recent studies suggested that BCa progression could be influenced by the tumor microenvironment (TME) [23, 24]. Several inflammatory immune cells in the TME may play important roles during BCa development and progression [23, 24]. Among those infiltrated immune cells, mast cells were frequently found in the BCa tissues [25]. Mast cells were previously known by their function in allergies and anaphylaxis, however, in recent years, evidence indicated mast cells may take part in fostering angiogenesis, tissue remodeling, and immunomodulation in various tumors [26, 27]. Therefore, mast cells in BCa may contribute to tumor angiogenesis and promote tumor progression [28]. However, the detailed mechanisms of how recruited mast cells may contribute to the BCa progression remain unclear.

Together, we have utilized several *in vitro* and *in vivo* strategies to demonstrate the recruited mast cells could enhance BCa cells invasion *via* stimulating the ERβ/CCL2/CCR2/EMT/MMP9 signal pathway.

## RESULTS

### BCa tissues recruit more mast cells than non-malignant tissues

**Figure 1:**
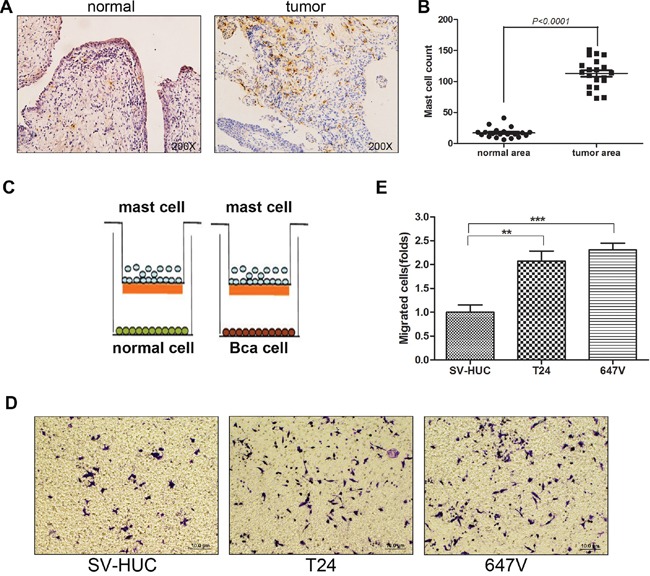
Bladder cancer tissues recruit more mast cells than non-malignant bladder tissues **A.** Immunohistochemical staining of tryptase as the cell marker to detect mast cells in human BCa and in adjacent non-malignant bladder tissues (mast cells are stained dark brown, original magnification × 200). **B.** Quantification of mast cell counts in BCa tissues and normal bladder tissues (mean ± SD of numbers of mast cells per five fields of view at × 200 magnification). **C.** Cartoon illustration of the mast cell migration assay. The insert upper chambers with 5 μm pore polycarbonate membrane were pre-coated with 10 ng/ml fibronectin. HMC-1 cells (1 × 10^5^) were placed in the inserted wells, BCa cells or non-malignant bladder epithelial cells (1 × 10^6^) were cultured in the bottom wells to assay the migration rate of mast cells. **D.** BCa cells promote mast cell migration. Mast cells (1 × 10^5^) were added in the upper chambers. We seeded non-malignant bladder SV-HUC cells and 2 different BCa cell lines, T24 and 647V (1 × 10^6^) in the bottom wells. After 4 hrs of incubation, the bottom sides of insert wells were fixed and stained to visualize the migrated mast cells. **E.** Quantitation data for migrated mast cells. Results were presented as mean ± SD. Statistical analysis was done by two-tailed Student's *t* test (**, *p* < 0.01; ***, *p* < 0.001).

Early studies reported that mast cells could be recruited to various tumors [26–28, 32]. We were interested in testing whether BCa tissues have a better capacity to recruit mast cells as compared to the surrounding non-malignant bladder tissues. We first applied IHC staining using tryptase, a marker to stain mast cells, in human BCa samples. Results showed that more infiltrated mast cells were found in BCa than in adjacent non-malignant bladder tissues (Figure 1A–1B).

To confirm these *in vivo* clinical data, we then applied the Boyden chamber migration system (Figure 1C) to assay the HMC-1 mast cell migration ability toward BCa T24 and 647V cells *vs*. non-malignant bladder SV-HUC cells. Consistently, the results showed T24 and 647V cells have a much better capacity to recruit mast cells as compared to SV-HUC cells (Figure 1D–1E). Together, both *in vivo* human clinical data and *in vitro* migration assay data proved that BCa tissues could recruit more mast cells than the surrounding non-malignant bladder tissues.

### Recruited mast cells could promote BCa cells invasion

**Figure 2:**
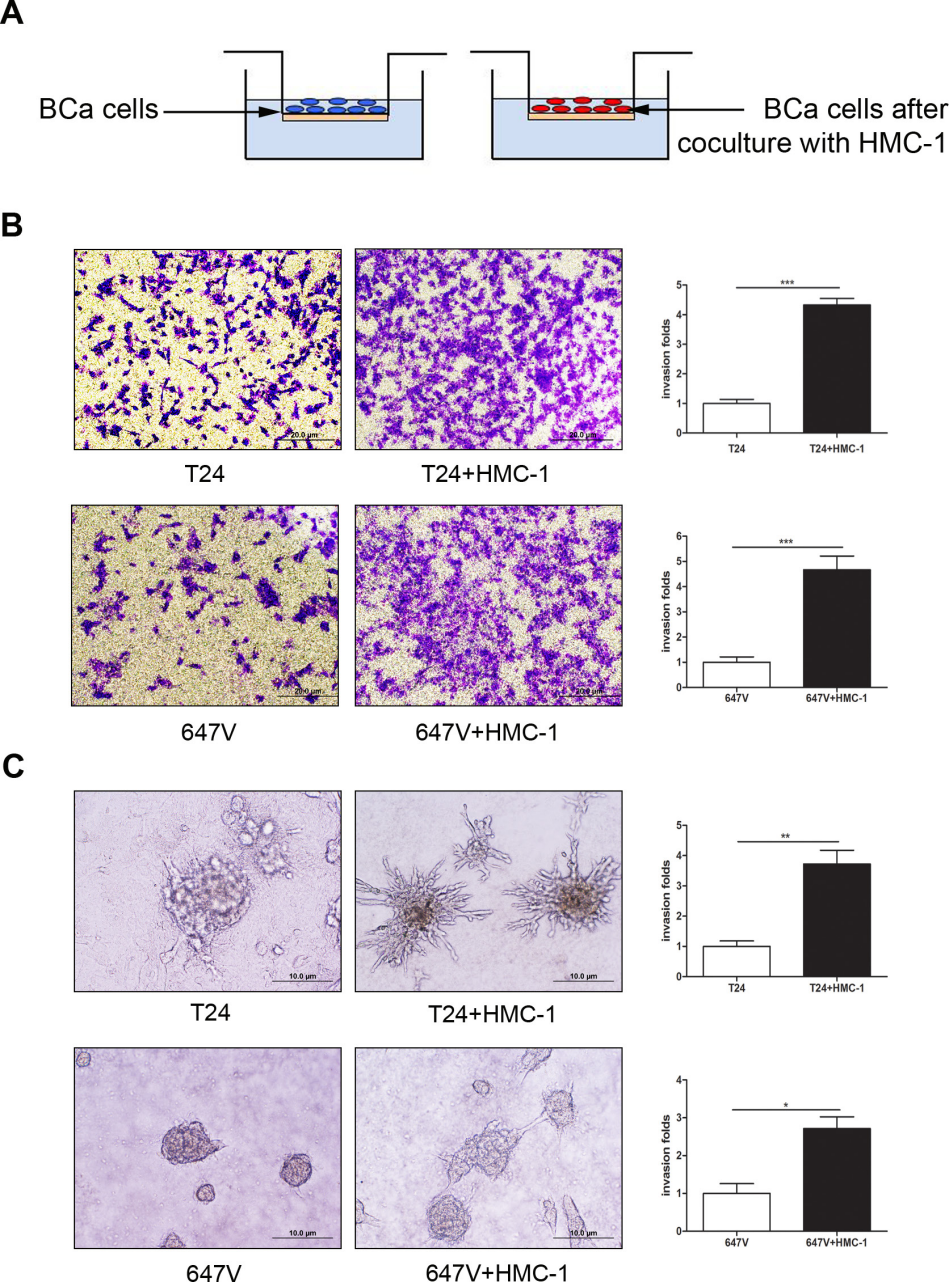
Recruited mast cells could promote BCa cells invasion **A.** The cartoon illustrates the invasion assay. BCa cells with or without co-culture with HMC-1 cells were seeded into the upper chambers (with 8 μm size pore and pre-coated with Matrigel) for 24 hrs to perform invasion assay. **B.** T24, and 647V cells were seeded in the upper wells to perform invasion assay and toluidine blue staining results showed BCa cells, after being co-cultures with HMC-1 cells, have a higher invasive capacity as compared to untreated BCa cells. **C.** 3D spheroid invasion assay. Representative micrograph of BCa cells grown on Matrigel for 10 days. The results showed HMC-1 co-cultured BCa cells grew into more dense sphere-like colonies with invasive projections emanating from the cells as compared with non-co-cultured BCa cells. Quantifications are shown in the right panels. *, *p* < 0.05;**, *p* < 0.01; ***, *p* < 0.001.

We then applied the chamber invasion assay in co-culture system (Figure 2A) to examine the consequences of increased infiltrating mast cells on BCa progression. We first treated HMC-1 mast cells with PMA to induce the mast cell differentiation and maturation. We then used these matured HMC-1 mast cells to co-culture with 2 different BCa cells (T24 and 647v) for 48 hrs and then to test the BCa cell capacity for invasion. As shown in Figure 2B, Boyden chamber invasion assay, both T24 and 647v BCa cells become more invasive after co-culture with mast cells.

We then applied the 3D invasion assay [33] to confirm the above findings. As shown in Figure 2C, in 3D culture, BCa cells grew into more structurally well-organized spheres, and formed more invasive projections surrounding the spheres of BCa cells that have been co-cultured with HMC-1 cells, which indicated BCa cells gained a better invasion capacity.

Together, results from Figure 2A–2C with two BCa cell lines and different invasion assays demonstrated that recruited mast cells could enhance the invasion capability of these BCa cells.

### Mechanism dissection by which the recruited mast cells could promote BCa cell invasion

**Figure 3:**
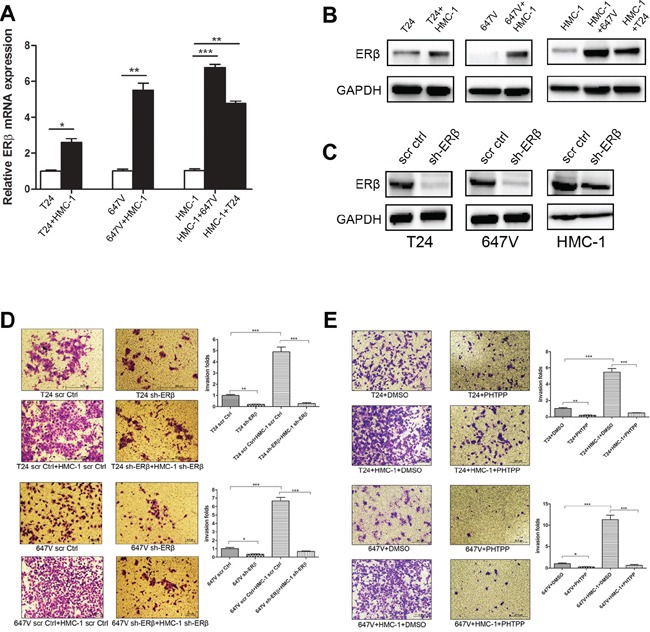
Recruited mast cells could promote BCa cell invasion *via* up-regulating ERβ **A.** Q-PCR results showed an increased ERβ expression in BCa cells (T24 and 647V) as well as in HMC-1 cells after co-culture. **B.** Western blot results showed ERβ protein expression increased both in BCa cells (T24, 647V) and in HMC-1 cells after co-culture. **C.** Validation of sh-ERβ knockdown efficiency in BCa and HMC-1 cells using Western blot. **D.** HMC-1 cell co-culture could not effectively promote the invasion in BCa cells with ERβ knockdown. We used lentiviral sh-ERβ to knock down ERβ expression in BCa cells, or in HMC-1 cells. Mast cells co-culture failed to promote invasion of BCa cells in the co-culture system with knockdown ERβ compared with scramble as control. **E.** Blocking ERβ with 10 μM PHTPP (a selective ERβ antagonist) could reverse mast cells-promoted BCa cell invasion in both T24 and 647V cells. Quantifications are shown in the right panels of (D) and (E) *, *p* < 0.05; **, *p* < 0.01; ***, *p* < 0.001.

To dissect the molecular mechanism why increased infiltrated mast cells could enhance BCa cells invasion, we have screened a group of genes that are reported to be associated with cancer cells invasion or BCa progression. Among those genes, we found that estrogen receptor beta (ERβ) mRNA is selectively increased in the BCa cells after being co-cultured with mast cells, HMC-1 (Figure 3A). Interestingly, the expression of ERβ was also increased in the co-cultured HMC-1 cells (Figure 3A). Earlier report using *in vitro* cell culture assay and *in vivo* carcinogen-induced BCa model indeed showed that ERβ could promote BCa metastasis [15]. Using Western blot, we further validated that recruited HMC-1 cells could increase ERβ expression at the protein levels in T24 and 647V BCa cells and the expression of ERβ protein in HMC-1 cells also increased after being co-cultured with BCa cells for 48 hours (Figure 3B).

We then applied the interruption approach to validate the importance of ERβ in mast cell-promoted BCa invasion. We used ERβ-shRNA to knock-down ERβ in BCa cells as well as HMC-1 cells, and results showed that suppressed ERβ could reverse mast cell-promoted BCa cell invasion in both T24 and 647V cells (Figure 3C–3D). Similar results were also obtained when we replaced the ERβ-shRNA with an ERβ specific antagonist, PHTPP, showing blocking ERβ with PHTPP could reverse mast cell-promoted BCa cell invasion in both T24 and 647V cells (Figure 3E).

Together, results from Figure 3 demonstrated that infiltrated mast cells could function through stimulating ERβ expression to promote the BCa cell invasion.

### Recruited mast cells enhanced BCa cell invasion *via* increasing ERβ-CCL2 signals

**Figure 4:**
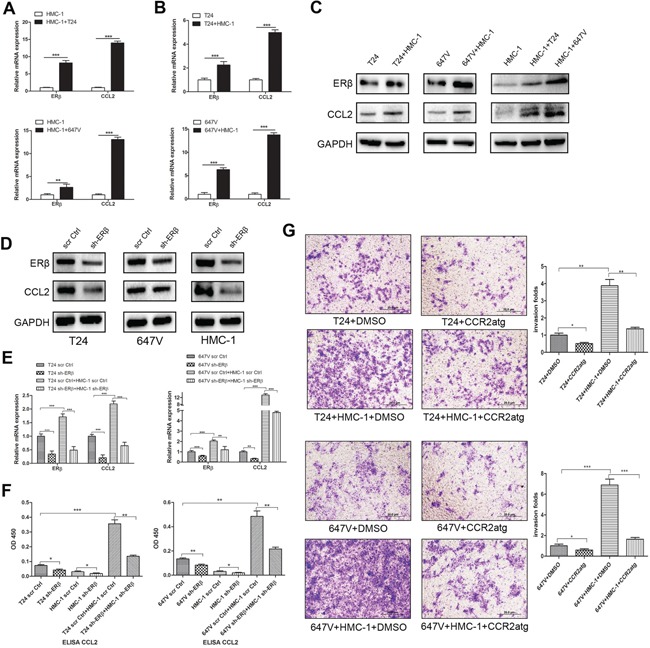
Recruited mast cells could increase ERβ and consequently up-regulate CCL2 signaling **A.** Q-PCR results showed CCL2 expression was significantly increased in mast cells after co-culture with T24 and 647V cells for 48 hrs. **B.** Q-PCR results showed CCL2 expression was significantly increased in T24 and 647V cells after co-culture with mast cells for 48 hrs. **C.** Western blot results showed ERβ and CCL2 protein expressions were increased in T24, 647V and HMC-1 cells after co-culture. **D.** Western Blot results showed ERβ knockdown could effectively decrease the expression of CCL2 in BCa cells as well as in mast cells. **E.** Q-PCR results showed that knocking-down ERβ could decrease the expression of CCL2 in BCa cells. Mast and BCa cell co-culture increased CCL2 expression level can be partially reversed by ERβ knockdown in BCa cells or in HMC-1 cells. **F.** ELISA assay showed that CCL2 was increased in conditional media (CM) collected after BCa cells co-culture with mast cells. The co-culture-increased CCL2 expression can be partially reversed by knocking down ERβ. **G.** Blocking CCL2 with CCR2 antagonist could at least partially reverse the mast cell-promoted BCa cell invasion in both T24 and 647v cells. *, *p* < 0.05; **, *p* < 0.01; ***p < 0.001.

To further dissect the mechanisms by which ERβ signals may be involved in the recruited mast cell-promoted BCa cell invasion, we then applied the focus array with most reported cytokines related to tumor metastasis by comparing gene profiles in mast cells co-culture with vs without BCa cells. We found a higher CCL2 expression in mast cells after co-culture with either BCa T24 or 647V cells (Figure 4A). Interestingly, we also found the expression of CCL2 increased in both T24 and 647V cells after co-culture with mast cells (Figure 4B). We further applied the Western Blot to confirm the mRNA data, and results showed the increase of both ERβ and CCL2 protein expressions either in BCa cells or in mast cells after being co-culture for 48 hours (Figure 4C).

Using interruption approaches with ERβ-shRNA to knock down ERβ, we found the decreased ERβ expression led to the suppressed CCL2 expression in BCa cells and in mast cells at mRNA and protein levels (Figure 4D). Consistently, we found the co-culture induced CCL2 expressions could be partially reversed by ERβ knockdown in those 2 BCa cell lines or in HMC-1 cells (Figure 4E). Importantly and supportively, ELISA assay data confirmed that the secreted CCL2 in conditioned media (CM) was increased after co-culturing mast cells and BCa cells, and this increased CCL2 could be reversed after adding ERβ-shRNA to knock down ERβ either in BCa cells, or in HMC-1 cells (Figure 4F). Furthermore, blocking CCL2 by CCR2 antagonist can also partly reverse mast cell-promoted BCa cell invasion in both T24 and 647V cells (Figure 4G).

Together, results from Figure 4 using multiple approaches in different BCa cells proved that recruited mast cells could enhance BCa cell invasion *via* promoting ERβ-CCL2 signals in BCa tumor environment.

### CCL2 could increase the epithelial-mesenchymal transition (EMT) signals to enhance BCa cell invasion

**Figure 5:**
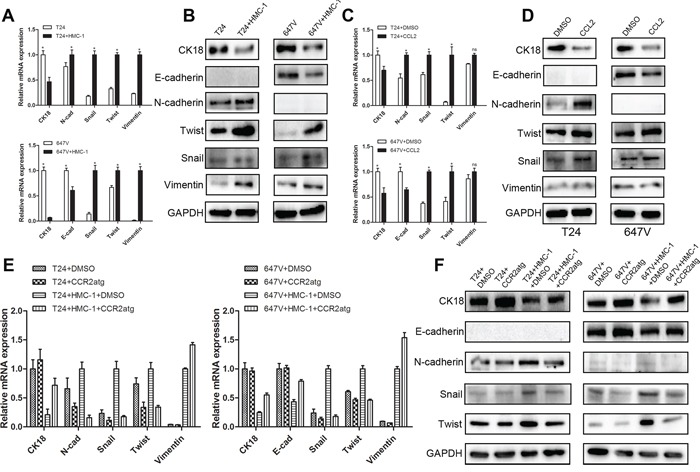
CCL2 could induce EMT signal pathways in BCa cells **A–B.** Q-PCR and Western blot results showed that epithelial markers decreased and mesenchymal markers increased in T24 and 647V cells after co-cultured with mast cells (*, *p* < 0.05). **C.** Q-PCR results showed that mRNA levels of EMT markers were up-regulated in T24 and 647V cells after treated with CCL2 (*, *p* < 0.05). **D.** Western Blot results showed that protein levels of EMT markers were increased in BCa cells after treated with CCL2. **E–F.** Q-PCR and Western blot results showed that CCR2 antagonist treatment could reverse the expressions of EMT markers in BCa cells after co-cultured with mast cells.

To further dissect the CCL2 downstream signals involving the promotion of the BCa cell invasion, we examined the potential linkage of CCL2 regulation on the EMT signal pathways as early studies [34] indicated that EMT is involved in the bladder tumor metastasis. It has been known that T24 cells were E-cadherin negative cells [35]. As shown in Figure 5A, we found that the E-cadherin is undetectable, the expression of CK18 (an epithelial marker) was decreased, and the mesenchymal markers, including N-cadherin, Twist, Snail and Vimentin, were significantly up-regulated in BCa T24 cells after co-culture with mast cells. In 647V cells, we obtained the consistent and supportive results showing that epithelial markers, including E-cadherin and CK18, which were drastically down-regulated, and mesenchymal markers, such as Twist, Snail and Vimentin, were dramatically up-regulated in co-cultured 647V cells. N-cadherin was undetected in 647V cells (Figure 5B).

Similar results were obtained when we added the CCL2 recombinant protein in the T24 and 647V cells except there was no significant change of Vimentin (Figure 5C–5D). Importantly, the interruption assay with addition of CCR2 antagonist, also resulted in partial reversal of these EMT markers (Figure 5E–5F).

Together, results from Figure 5 suggested that CCL2 could function through stimulating EMT to alter the BCa cell invasion.

### EMT enhanced BCa cell invasion by up-regulation of the MMP9 signal

**Figure 6:**
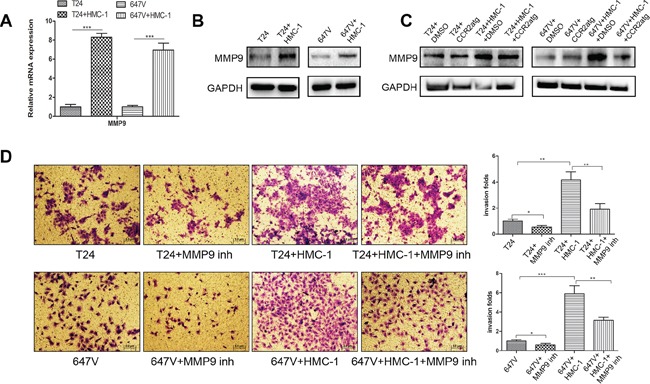
EMT could function via increasing MMP9 to enhance BCa cell invasion **A.** Q-PCR results show mRNA of MMP9 increased in BCa cells after 48 hour-co-culture with mast cells. **B.** Western Blot results showed the protein of MMP9 increased in BCa cells after co-cultured with mast cells. **C.** Western blot results showed that interruption of CCL2 signal by adding CCR2 antagonist resulted in partial reversal of the increased MMP9 expression in both T24 and 647V cells after co-culture with mast cells. **D.** Blocking MMP9 with MMP9 inhibitor (inh) can partly reverse mast cell-promoted T24 and 647V cells invasion. *, *p* < 0.05; **, *p* < 0.01; ***p < 0.001.

From a focus array with different BCa metastasis-related genes [36], we found MMP9 expression was specifically enhanced in these two BCa cells after co-culture with mast cells at both mRNA (Figure 6A) and protein levels (Figure 6B). This result is in agreement with an early report showing EMT could function through MMP9 to alter the invasion of skin cancer cells [37]. Interrupting the pathway by adding CCR2 antagonist also resulted in partial reversal of the increased expression of MMP9 in both T24 and 647V cells after co-culture with mast cells (Figure 6C). Importantly, using the interruption assay with the MMP9 inhibitor, we demonstrated that blocking MMP9 could partially reverse the mast cell-enhanced BCa cells invasion (Figure 6D).

Together, results from Figure 6 and above Figures 1–5 suggest the recruited mast cells could function through increasing ERβ/CCL2/CCR2/EMT/MMP9 signals to enhance the BCa cell invasion using *in vitro* cell system.

### Recruited mast cells promoted BCa metastasis *via* stimulating ERβ/CCL2/CCR2/EMT/MMP9 signals in the *in vivo* BCa model

**Figure 7:**
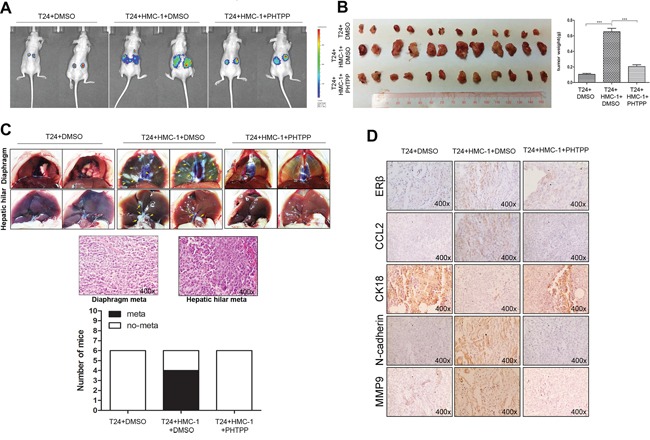
Mast cells promoted BCa metastasis via up-regulating ERβ/CCL2/CCR2/EMT/MMP9 signals *in vivo* **A.** Orthotopic xenografts of T24-Luc cells (1 × 10^6^) with or without 1 × 10^5^ mast HMC-1 cells under bilateral renal capsules of the female nude mice. The group with co-implanted mast cells was equally divided into two groups for DMSO or PHTPP intraperitoneal injection every other day. Once per week the IVIS Imaging was used to monitor tumor progression and metastasis. **B.** Tumor burden comparison between different tumor groups. The mice were sacrificed 4 weeks after treatments, and the primary tumor sizes in each group was observed and weighed. Quantifications are shown at the right panel. (***, *p* < 0.001). **C.** Metastasis rate comparison between different tumor groups. The metastatic tumors in the diaphragm and hepatic hilar area were counted, and confirmed by H&E histology staining after sacrifice. **D.** IHC staining confirmed the increased expressions of ERβ, CCL2, N-cadherin and MMP9, and the decreased expression of CK18 in mast cells co-implanted BCa tumors, and the ERβ antagonist PHTPP treatment can reverse these changes.

To confirm the above *in vitro* data showing the mast cell-enhanced BCa cell invasion *via* modulation of ERβ/CCL2/CCR2/EMT/MMP9 signals using the *in vivo* mouse BCa model, we transduced T24 cells with lentiviral-luciferase plasmid (T24-Luc) and then selected positive stable cells to expand. We orthotopically xenografted these T24-Luc cells (1 × 10^6^) with or without co-implanting 1 × 10^5^ mast HMC-1 cells under the bilateral renal capsules of female nude mice. Two weeks of tumor implantation, the mice with co-implant of mast and BCa cells were randomly divided into two groups for DMSO and PHTPP intraperitoneal injection every other day. After 4 weeks of treatments, we applied *in vivo* imaging system (IVIS), an infrared fluorescent detection method, to monitor tumor progression in live animals. The results revealed that the mice with co-implant of T24-Luc and HMC-1 cells had more metastatic luminescence signals located at distant organs compared to the mice implanted with T24-Luc cells only. There were 4 out of 6 mice showing such metastatic foci in the co-implanted group and no metastasis was found in mice implanted with T24-Luc cell only. More importantly, the treatment with the selective ERβ antagonist, PHTPP, could effectively reverse mast cell-promoted BCa metastasis with no mice showing the metastatic foci in this PHTPP treated group (Figure 7A and 7C). Interestingly, we also found T24-Luc and HMC-1 co-implanted group had increased growth rates and tumor weights compared to T24-Luc only group, while PHTPP treatment could result in much less tumor growth and tumor weights compared to co-implanted BCa group with mock treatment (Figure 7B).

Furthermore, H&E staining of those metastatic foci confirmed that those are indeed BCa cells, including those in the diaphragm and hepatic hilar regions (Figure 7C). Supportively, IHC staining results of ERβ, CCL2, CK18, N-cadherin and MMP9 were also in agreement with *in vitro* results showing mast cells could enhance BCa cell invasion *via* increasing ERβ/CCL2/CCR2/EMT/MMP9 signals *in vivo* (Figure 7D).

**Figure 8:**
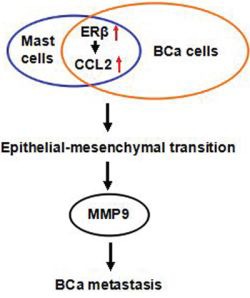
Cartoon illustration of mast cells-enhanced BCa invasion via increasing ERβ/CCL2/EMT/MMP9 signals in the tumor microenvironment. The red arrows indicate an increase in the ERβ and CCL2

Together, our data showed the infiltrated mast cells in the tumor microenvironment enhance BCa metastasis *via* stimulating ERβ/CCL2/CCR2 EMT/MMP9 signals both *in vitro* and *in vivo* (Figure 8). These results could lead to the development of a new potential therapeutic approach by targeting the identified signaling to better battle BCa metastasis.

## DISCUSSION

Epidemiological studies of BCa show that there is a gender difference in incidence with nearly three times more BCa in men than in women. This higher BCa incidence in males whereas less survival rate in female BCa patients indicated the complication of sex hormone signals, suggesting that both androgen and estrogen may play differential roles in the BCa incidence and invasion [38, 39]. Earlier studies found that ERβ is the predominant subtype of ER expressed in the bladder epithelium and smooth muscle of rats and mice [40, 41]. Recently, studies found the expression of ERβ, and not ERα, was detected in most of BCa samples with increased expression in higher stages and grades [14, 18].

Previous reports suggested that mast cell numbers were increased in high-grade BCa as compared to low-grade BCa [25, 42]. Additional report showed that the infiltrated mast cells could contribute to the tumor angiogenesis [28]. Consistent with these roles, here we demonstrated BCa could recruit more mast cells compared to normal urothelial cells with consequences to enhance BCa cell invasion *via* up-regulating the ERβ signals. This conclusion also strengthens the conclusion of an early report showing that inhibition of ERβ with ERβ-shRNA could suppress BCa cell invasion [15]. For the first time, our study proves that the tumor microenvironment mast cells could stimulate the ERβ/CCL2 signals, and consequently leads to an increased BCa cell invasion.

More importantly, our data further showed that increased CCL2 in CM from the co-culture of mast cells and BCa cells indeed plays important roles in promoting BCa cell invasion. The previous reported results suggest that CCL2 is a key player for tumor progression [43–45], and urinary CCL3 levels could be linked to the tumor stage, grade and distant metastasis [46–48], and patients with stages T2–T4 BCa have a three- to four-fold higher mean CCL2 concentration in their urine than those with T1 stage tumors [47]. Additional reported data suggested the potential importance of CCL2 and EMT signals in BCa development. For example, suppressing CCL2/CCR2 signals could reverse the paxillin-promoted migration and invasion of BCa cells [49]. Additional reports presented results showing that CCL2 could promote EMT in various tumors [45, 50, 51], and tumors undergoing the EMT may self-produce higher amounts of MMP9 [37, 52]. In the present study, we provided the first evidence to link the ERβ roles to the CCL2 expression in BCa cell invasion and suppressing the CCL2/CCR2 signals could decrease the ERβ effects on BCa cell invasion. Importantly, we further proved that the ERβ could function via up-regulating CCL2/CCR2 signals to increase EMT/MMP9 signals.

In summary, our study has identified and proven that infiltrated mast cells could enhance BCa metastasis via stimulating the ERβ/CCL2/CCR2/EMT/MMP9 signaling pathway in the BCa tumor microenvironment. Targeting this newly identified ERβ/CCL2/CCR2/EMT/MMP9 axis may help to develop alternative new therapies to suppress the BCa metastasis.

## MATERIALS AND METHODS

### Patient materials

Tumor specimens and adjacent normal urothelial tissues were collected from a total of 20 patients undergoing transurethral resection for bladder transitional cell carcinoma at the Huazhong University of Science and Technology affiliated Tongji Hospital. All specimens were obtained on the basis of their availability for research purposes and under a protocol approved by the local medical ethics committee of Tongji Medical Hospital. Written consent was obtained from the patients prior to the study. Patients with newly diagnosed BCa and who had no history of prior BCa surgery or any type of therapy treatment were included. The stage T1-T2 BCa tissues were also included in this study.

### Cell culture

T24 and SV-HUC cell lines were purchased from the American Type Culture Collection (ATCC, Manassas, VA). 647V cell line was purchased from German Collection of Microorganisms and Cell Cultures. Human mast cell line HMC-1 was a gift from Professor John Frelinger of the Eye institute of the University of Rochester Medical Center (Rochester, NY).

The SV-HUC cell line was established by transformation of normal ureter tissue with SV40 virus [29]. The 647V cell line was established from the primary transitional cell carcinoma (TCC) and reported by Elliott, A.Y. in 1976 [30]. The original report did not specifically describe the stage of TCC used to establish the 647V cell line. The T24 cell line was established from urinary carcinoma grade III in an 82-year old Swedish female with a long-term diagnosed urinary bladder popilomatoris [31]. Those 2 BCa cell lines have been extensively used in many BCa related studies. Based on the epithelial and stromal marker characterization, those two BCa cells are not the most malignant or invasive type of BCa cells. Therefore, they are suitable to characterize the effects of mast cells-promoted BCa invasion.

T24, 647V cells and SV-HUC cells were cultured in Dulbecco's Modified Eagle Media (DMEM) supplemented with 10% fetal bovine serum (FBS). HMC-1 cells were cultured in Iscove's modified Dulbecco's media (IMDM) supplemented with 10% heat inactivated FBS, 2 mM L-glutamine, 100 IU/mL penicillin and 50 μg/mL streptomycin. Cells were maintained in a humidified 5% CO_2_ environment at 37°C. When indicated, HMC-1 cells were exposed to the differentiation reagent, phorbol 12-myristate 13-acetate (PMA, Sigma-Aldrich), at 20 ng/ml for 3 days. For the remainder of this study, PMA treated HMC-1 cells were considered mature (differentiated) and untreated HMC-1 cells were considered immature (undifferentiated).

### Reagents and materials

Anti-GAPDH and anti-ERβ antibodies were purchased from Santa Cruz Biotechnology (CA, USA). Anti-MMP9, anti-E-cadherin, anti-Snail, anti-Twist and anti-MCP1 (CCL2) antibodies were from Abcam (MA, USA). Anti-CK18, anti-N-cadherin and anti-Vimentin antibodies were from Cell Signaling Technology (Boston, MA USA). Anti-mouse/rabbit second antibody for western blot was from Invitrogen (Carlsbad, CA, USA). CCR2 antagonist was purchased from Santa Cruz Biotechnology and was adjusted to a final concentration of 20 nM. Human CCL2 (MCP-1) recombinant protein was purchased from Affymetrix eBioscience (Santa Clara, CA, USA) and was adjusted to a final concentration of 10 nM. MMP9 inhibitor was purchased from ABGen and was adjusted to a final concentration of 5 μg/ml.

### Mast cell HMC-1 recruitment/migration assay

Mast cell migration was examined using a 24-well transwell system. Briefly, 1 × 10^6^ BCa cells or non-malignant SV-HUC cells were plated into the lower chambers of the transwells. The upper and lower chambers were separated by a 5 μm-polycarbonate filter coated with fibronectin (10 μg/ml, sc-29011, Santa Cruz) and dried for 1 hour in the hood. 1 × 10^5^ HMC-1 cells were then placed in the upper chambers. The chambers were incubated for 4 hours at 37°C to allow mast cells to migrate through the fibronectin. Filters were then scraped, washed, fixed with cold methanol, and stained with 1% toluidine blue. Cell migration was measured by counting the number of mast cells attached to the lower surface of the filter. Each assay was tested in triplicate. The results were expressed as the average of the number of migrating cells from upper chambers.

### Cell invasion assay

Cell invasion assays were performed using matrigel-coated transwell system. The upper chambers have membranes with 8 μm pore size and were pre-coated with diluted matrigel (BD Biosciences, Sparks, MD). Before performing invasion assays, BCa cells were co-cultured with mature mast cells for 48 hours. The mast cell-co-cultured BCa cells (1 × 10^5^, in serum free media) were seeded in the upper chambers, and 10% serum containing media were plated in the lower chambers. After 24 hours of incubation, invaded cells on the underside of the trans-well membrane were fixed and stained with 1% toluidine blue. The invaded cells were counted and averaged from six random fields. Data are presented as mean ± SD of results from 5 representative fields. Each experiment has been performed 3 independent times, each with triplicate data points.

### 3D invasion assay

We added 40 μl of Matrigel evenly to each well of 8-well glass chamber slide (at 50 μl/cm^2^). Then, the slides were placed in the cell culture incubator for 15–20 min to allow the Matrigel to solidify. BCa cells (1 × 10^4^) were plated into each well with media containing 5% Matrigel and 10 ng/ml EGF. Media (2.5% Matrigel and 5.0 ng/ml EGF) was changed every 4 days. The formations of 3D spherical protruding structures between BCa cells were photographed at 2-day intervals for a total of 10 days. Six different random fields under 200× microscope were chosen, and the numbers of protruding structures were counted in each field. Quantitation data were presented as mean ± SD of results from 5 representative fields. Each experiment has been performed 3 independent times, each with triplicate data points.

### RNA extraction, and quantitation by real-time PCR

Total RNAs were isolated using Trizol reagent (Invitrogen, Grand Island, NY). One μg of total RNA was subjected to reverse transcription using Superscript III transcriptase (Invitrogen, Grand Island, NY). Quantitative real-time PCR (Q-RT-PCR) assay was conducted using a Bio-Rad CFX96 system with SYBR green to determine the mRNA expression level of a gene of interest. Expression levels were normalized to the expression of GAPDH RNA. (All primers used are listed in Supplementary Data Table S1).

### Western blot analysis

Cells were lysed in RIPA buffer, proteins (20 μg) were separated on 8–10% SDS/PAGE gel, and then transferred onto PVDF membranes (Millipore, Billerica, MA). After blocking membranes, they were incubated with appropriate dilutions of specific primary antibodies as indicated. The membrane blots were then incubated with horseradish peroxidase (HRP)-conjugated secondary antibodies and visualized using ECL system (Thermo Fisher Scientific, Rochester, NY).

### ELISA assay

Conditioned media (CM) was collected from T24 cells with short hairpin RNA for scramble sequence (T24 scr) and short hairpin RNA for ERβ (T24 sh-ERβ), 647V cells (scr and sh-ERβ), or from co-cultures of T24 cells (scr and sh-ERβ) +HMC-1 cells, or 647V cells (scr and sh-ERβ) +HMC-1 cells for 48 hours. CM was used for the detection of CCL2 by human CCL2 ELISA kits (R&D Systems) according to the manufacturer's instructions.

### Lentivirus packaging and cell transfection

Sh-ERβ was constructed into the pLKO.1 lentiviral vector as reported previously [15]. The pLKO.1 sh-ERβ together with package and envelope plasmids, psPAX2 and pMD2G, were co-transfected into 293T cells for 48 hours to produce the ERβ shRNA lentivirus particle soup. Lentivirus soup was then collected and frozen at −80°C for later use in transduction of BCa cells.

### *In vivo* BCa metastasis rate and anti-estrogen treatment

Female (7–8 weeks old) nude mice were purchased from NCI. To monitor the tumor metastasis by *In Vivo* Image System (IVIS), T24 cells were engineered to express luciferase reporter gene (pCDNA3.0-luciferase) by stable transfection and the positive stable clones were selected and expanded in culture (T24-Luc). 6 mice were injected with T24-Luc cells (1 × 10^6^) mixed with Matrigel at 1:1. Another 12 mice were co-injected with T24-Luc (9 × 10^5^) and HMC-1 (1 × 10^5^) cells mixed with matrigel at 1:1 under the bilateral renal capsules. One week after implantation, these 12 co-implanted mice were randomly divided into two groups for treatment with PHTPP or mock DMSO. PHTPP (10 μl of 10 mM PHTPP per mouse) or mock DMSO was mixed with 90 μl of sesame oil and injected-intraperitoneally every other day for 4 weeks. BCa metastasis was monitored once per week using a Fluorescent Imager (IVIS Spectrum, Caliper Life Sciences, Hopkinton, MA). After a final IVIS imaging, the mice were sacrificed, primary and metastatic tumors were collected for H&E and IHC staining.

### Histological staining

The specimens were fixed in 4% para-formaldehyde, embedded in paraffin, and then sectioned at a thickness of 5 μm. For the H&E staining, after de-waxing and rehydration, tissue sections were stained in haematoxylin for 5 min and washed by running tap water for 5 min. Then the sections were stained in eosin for 30 sec, dehydrated, and mounted by routine methods.

### Immunohistochemical staining

After dewaxing and rehydration, sections were pretreated with 10% H_2_O_2_. After blocking with 5% BSA, all sections were incubated with the primary antibodies (as indicated) at 4°C overnight. The primary antibody was recognized by the biotinylated secondary antibody (Vector), and visualized by VECTASTAIN ABC peroxidase system and peroxidase substrate DAB kit (Vector). The primary antibodies used were: rabbit anti-ERβ (Abcam, 12C8), rabbit anti-MMP9 (Cell Signaling), anti-CCL2 (CCL2 is also named as MCP-1) and anti-mast cell tryptase (Abcam), mouse anti-CK18 and rabbit anti-N-cadherin (Cell Signaling).

### Statistics

All statistical analyses were performed with SPSS 16.0 (SPSS Inc, Chicago, IL). The data values were presented as the mean ± SD. Differences in mean values between two groups were analyzed by two-tailed Student's *t* test. *p* ≤ 0.05 was considered statistically significant.

## SUPPLEMENTARY TABLES



## References

[1] Siegel R, Ma J, Zou Z, Jemal A (2014). Cancer statistics, 2014. CA Cancer J Clin.

[2] Dougherty D.W, Gonsorcik V.K, Harpster L.E, Trussell J, Drabick J.J (2009). Superficial bladder cancer metastatic to the lungs: two case reports and review of the literature. Urology.

[3] Lindberg M.K, Weihua Z, Andersson N, Moverare S, Gao H, Vidal O, Erlandsson M, Windahl S, Andersson G, Lubahn D.B, Carlsten H, Dahlman-Wright K, Gustafsson J.A (2002). Estrogen receptor specificity for the effects of estrogen in ovariectomized mice. J Endocrinol.

[4] Couse J.F, Dixon D, Yates M, Moore A.B, Ma L, Maas R, Korach K.S (2001). Estrogen receptor-alpha knockout mice exhibit resistance to the developmental effects of neonatal diethylstilbestrol exposure on the female reproductive tract. Dev Biol.

[5] Spencer-Segal J.L, Tsuda M.C, Mattei L, Waters E.M, Romeo R.D, Milner T.A, McEwen B.S, Ogawa S (2011). Estradiol acts via estrogen receptors alpha and beta on pathways important for synaptic plasticity in the mouse hippocampal formation. Neuroscience.

[6] Hill L, Jeganathan V, Chinnasamy P, Grimaldi C, Diamond B (2010). Differential roles of estrogen receptors alpha and beta in control of B-cell maturation and selection. Mol Med.

[7] Bjornstrom L, Sjoberg M (2005). Mechanisms of estrogen receptor signaling: convergence of genomic and nongenomic actions on target genes. Mol Endocrinol.

[8] Leung Y.K, Mak P, Hassan S, Ho S.M (2006). Estrogen receptor (ER)-beta isoforms: a key to understanding ER-beta signaling. Proc Natl Acad Sci U S A.

[9] Prins G.S, Korach K.S (2008). The role of estrogens and estrogen receptors in normal prostate growth and disease. Steroids.

[10] Chen M, Hsu I, Wolfe A, Radovick S, Huang K, Yu S, Chang C, Messing E.M, Yeh S (2009). Defects of prostate development and reproductive system in the estrogen receptor-alpha null male mice. Endocrinology.

[11] Ricke W.A, McPherson S.J, Bianco J.J, Cunha G.R, Wang Y, Risbridger G.P (2008). Prostatic hormonal carcinogenesis is mediated by in situ estrogen production and estrogen receptor alpha signaling. FASEB J.

[12] Vitkus S, Yeh C.R, Lin H.H, Hsu I, Yu J, Chen M, Yeh S (2013). Distinct function of estrogen receptor alpha in smooth muscle and fibroblast cells in prostate development. Mol Endocrinol.

[13] Song W, Yeh C.R, He D, Wang Y, Xie H, Pang S.T, Chang L.S, Li L, Yeh S (2015). Infiltrating neutrophils promote renal cell carcinoma progression via VEGFa/HIF2alpha and estrogen receptor beta signals. Oncotarget.

[14] Sonpavde G, Okuno N, Weiss H, Yu J, Shen S.S, Younes M, Jian W, Lerner S.P, Smith C.L (2007). Efficacy of selective estrogen receptor modulators in nude mice bearing human transitional cell carcinoma. Urology.

[15] Hsu I, Yeh C.R, Slavin S, Miyamoto H, Netto G.J, Tsai Y.C, Muyan M, Wu X.R, Messing E.M, Guancial E.A, Yeh S (2014). Estrogen receptor alpha prevents bladder cancer via INPP4B inhibited akt pathway in vitro and in vivo. Oncotarget.

[16] Hsu I, Vitkus S, Da J, Yeh S (2013). Role of oestrogen receptors in bladder cancer development. Nature Reviews Urology.

[17] Yeh C.R, Hsu I, Song W, Chang H, Miyamoto H, Xiao G.Q, Li L, Yeh S (2015). Fibroblast ERalpha promotes bladder cancer invasion via increasing the CCL1 and IL-6 signals in the tumor microenvironment. Am J Cancer Res.

[18] Shen S.S, Smith C.L, Hsieh J.T, Yu J, Kim I.Y, Jian W, Sonpavde G, Ayala G.E, Younes M, Lerner S.P (2006). Expression of estrogen receptors-α and -β in bladder cancer cell lines and human bladder tumor tissue. Cancer.

[19] Kontos S, Kominea A, Melachrinou M, Balampani E, Sotiropoulou-Bonikou G (2010). Inverse expression of estrogen receptor-β and nuclear factor-κB in urinary bladder carcinogenesis. International journal of urology.

[20] Tuygun C, Kankaya D, Imamoglu A, Sertcelik A, Zengin K, Oktay M, Sertcelik N (2011). Sex-specific hormone receptors in urothelial carcinomas of the human urinary bladder: a comparative analysis of clinicopathological features and survival outcomes according to receptor expression.

[21] Miyamoto H, Yao J.L, Chaux A, Zheng Y, Hsu I, Izumi K, Chang C, Messing E.M, Netto G.J, Yeh S (2012). Expression of androgen and oestrogen receptors and its prognostic significance in urothelial neoplasm of the urinary bladder. BJU Int.

[22] Kauffman E.C, Robinson B.D, Downes M, Marcinkiewicz K, Vourganti S, Scherr D.S, Gudas L.J, Mongan N.P (2013). Estrogen receptor-β expression and pharmacological targeting in bladder cancer. Oncology reports.

[23] Eruslanov E, Daurkin I, Vieweg J, Daaka Y, Kusmartsev S (2011). Aberrant PGE(2) metabolism in bladder tumor microenvironment promotes immunosuppressive phenotype of tumor-infiltrating myeloid cells. Int Immunopharmacol.

[24] Chi L.J, Lu H.T, Li G.L, Wang X.M, Su Y, Xu W.H, Shen B.Z (2010). Involvement of T helper type 17 and regulatory T cell activity in tumour immunology of bladder carcinoma. Clin Exp Immunol.

[25] Kim J.H, Kang Y.J, Kim D.S, Lee C.H, Jeon Y.S, Lee N.K, Oh M.H (2011). The relationship between mast cell density and tumour grade in transitional cell carcinoma of the bladder. J Int Med Res.

[26] Soucek L, Lawlor E.R, Soto D, Shchors K, Swigart L.B, Evan G.I (2007). Mast cells are required for angiogenesis and macroscopic expansion of Myc-induced pancreatic islet tumors. Nat Med.

[27] Gounaris E, Erdman S.E, Restaino C, Gurish M.F, Friend D.S, Gounari F, Lee D.M, Zhang G, Glickman J.N, Shin K, Rao V.P, Poutahidis T, Weissleder R (2007). Mast cells are an essential hematopoietic component for polyp development. Proc Natl Acad Sci U S A.

[28] Sari A, Calli A, Cakalagaoglu F, Altinboga A.A, Bal K (2012). Association of mast cells with microvessel density in urothelial carcinomas of the urinary bladder. Ann Diagn Pathol.

[29] Christian B.J, Loretz L.J, Oberley T.D, Reznikoff C.A (1987). Characterization of human uroepithelial cells immortalized in vitro by simian virus 40. Cancer research.

[30] Elliott A.Y, Bronson D.L, Stein N, Fraley E.E (1976). In vitro cultivation of epithelial cells derived from tumors of the human urinary tract. Cancer research.

[31] Bubenik J, Barešová M, Viklický V, Jakoubkova J, Sainerova H, Donner J (1973). Established cell line of urinary bladder carcinoma (T24) containing tumour-specific antigen. International journal of cancer.

[32] Oldford S.A, Marshall J.S (2015). Mast cells as targets for immunotherapy of solid tumors. Mol Immunol.

[33] Cai J, Guan H, Fang L, Yang Y, Zhu X, Yuan J, Wu J, Li M (2013). MicroRNA-374a activates Wnt/beta-catenin signaling to promote breast cancer metastasis. J Clin Invest.

[34] Yun S.J, Kim W.J (2013). Role of the epithelial-mesenchymal transition in bladder cancer: from prognosis to therapeutic target. Korean J Urol.

[35] Bindels E.M, Vermey M, De Both N.J, van der Kwast T.H (2001). Influence of the microenvironment on invasiveness of human bladder carcinoma cell lines. Virchows Arch.

[36] Slaton J.W, Millikan R, Inoue K, Karashima T, Czerniak B, Shen Y, Yang Y, Benedict W.F, Dinney C.P (2004). Correlation of metastasis related gene expression and relapse-free survival in patients with locally advanced bladder cancer treated with cystectomy and chemotherapy. J Urol.

[37] Coussens L.M, Tinkle C.L, Hanahan D, Werb Z (2000). MMP-9 supplied by bone marrow-derived cells contributes to skin carcinogenesis. Cell.

[38] Siegel R, Ward E, Brawley O, Jemal A (2011). Cancer statistics, 2011: the impact of eliminating socioeconomic and racial disparities on premature cancer deaths. CA Cancer J Clin.

[39] Pfister C (2001). New trends for optimal management of bladder tumors. J Urol.

[40] Makela S, Strauss L, Kuiper G, Valve E, Salmi S, Santti R, Gustafsson J.A (2000). Differential expression of estrogen receptors alpha and beta in adult rat accessory sex glands and lower urinary tract. Mol Cell Endocrinol.

[41] Savolainen S, Santti R, Streng T, Gustafsson J.A, Harkonen P, Makela S (2005). Sex specific expression of progesterone receptor in mouse lower urinary tract. Mol Cell Endocrinol.

[42] Serel T.A, Soyupek S, Candir O (2004). Association between mast cells and bladder carcinoma. Urol Int.

[43] Deshmane S.L, Kremlev S, Amini S, Sawaya B.E (2009). Monocyte chemoattractant protein-1 (MCP-1): an overview. J Interferon Cytokine Res.

[44] Raman D, Baugher P.J, Thu Y.M, Richmond A (2007). Role of chemokines in tumor growth. Cancer letters.

[45] Izumi K, Fang L.Y, Mizokami A, Namiki M, Li L, Lin W.J, Chang C (2013). Targeting the androgen receptor with siRNA promotes prostate cancer metastasis through enhanced macrophage recruitment via CCL2/CCR2-induced STAT3 activation. EMBO Mol Med.

[46] Narter K.F, Agachan B, Sozen S, Cincin Z.B, Isbir T (2010). CCR2-64I is a risk factor for development of bladder cancer. Genet Mol Res.

[47] Amann B, Perabo F.G, Wirger A, Hugenschmidt H, Schultze-Seemann W (1998). Urinary levels of monocyte chemo-attractant protein-1 correlate with tumour stage and grade in patients with bladder cancer. Br J Urol.

[48] Vazquez-Lavista L.G, Lima G, Gabilondo F, Llorente L (2009). Genetic association of monocyte chemoattractant protein 1 (MCP-1)-2518 polymorphism in Mexican patients with transitional cell carcinoma of the bladder. Urology.

[49] Chiu H.Y, Sun K.H, Chen S.Y, Wang H.H, Lee M.Y, Tsou Y.C, Jwo S.C, Sun G.H, Tang S.J (2012). Autocrine CCL2 promotes cell migration and invasion via PKC activation and tyrosine phosphorylation of paxillin in bladder cancer cells. Cytokine.

[50] Low-Marchelli J.M, Ardi V.C, Vizcarra E.A, van Rooijen N, Quigley J.P, Yang J (2013). Twist1 induces CCL2 and recruits macrophages to promote angiogenesis. Cancer Res.

[51] Lee S.H, Kang H.Y, Kim K.S, Nam B.Y, Paeng J, Kim S, Li J.J, Park J.T, Kim D.K, Han S.H, Yoo T.H, Kang S.W (2012). The monocyte chemoattractant protein-1 (MCP-1)/CCR2 system is involved in peritoneal dialysis-related epithelial-mesenchymal transition of peritoneal mesothelial cells. Lab Invest.

[52] Pittoni P, Colombo M.P (2012). The dark side of mast cell-targeted therapy in prostate cancer. Cancer Res.

