# Supplementary material for: Recruited mast cells in the tumor microenvironment enhance bladder cancer metastasis via modulation of ERβ/CCL2/CCR2 EMT/MMP9 signals
Source: Oncotarget. 2015 Nov 5;7(7):7842–55. doi: 10.18632/oncotarget.5467 (PMC4884958; doi:10.18632/oncotarget.5467)
Supplement: Supplementary file 1 [file oncotarget-07-7842-s001.pdf]

SUPPLEMENTARY TABLE

Supplementary Table S1: Primers for qPCR

Target genes	Sense	Antisense
ERβ	AGCACGGCTCCATATACATACC	TGGACCACTAAAGGAGAAAGGT
CCL2	CAGCCAGATGCAATCAATGCC	TGGAATCCTGAACCCACTTCT
CK18	GGCATCCAGAACGAGAAGGAG	ATTGTCCACAGTATTTGCGAAGA
E-cadherin	CGAGAGCTACACGTTCACGG	GGGTGTCGAGGGAAAAATAGG
N-cadherin	TTTGATGGAGGTCTCCTAACACC	ACGTTTAACACGTTGGAAATGTG
Snail	ACTGCGACAAGGAGTACACC	GAGTGCGTTTGCAGATGGG
Twist	GCCTAGAGTTGCCGACTTATG	TGCGTTTCCTGTTAAGGTAGC
Vimentin	GACGCCATCAACACCGAGTT	CTTTGTCGTTGGTTAGCTGGT
MMP9	TGTACCGCTATGGTTACACTCG	GGCAGGGACAGTTGCTTCT
GAPDH	TGTGGGCATCAATGGATTTGG	ACACCATGTATTCCGGGTCAAT
